# Optically Transparent Microwave Polarizer Based On Quasi-Metallic Graphene

**DOI:** 10.1038/srep17083

**Published:** 2015-11-25

**Authors:** Marco Grande, Giuseppe Valerio Bianco, Maria Antonietta Vincenti, Domenico de Ceglia, Pio Capezzuto, Michael Scalora, Antonella D’Orazio, Giovanni Bruno

**Affiliations:** 1 Dipartimento di Ingegneria Elettrica e dell’Informazione, Politecnico di Bari, Bari, 70125, Italy; 2 Istituto di Nanotecnologia – CNR-NANOTEC, Bari, 70126, Italy; 3 National Research Council, Charles M. Bowden Research Center, RDECOM, Redstone Arsenal, Alabama 35898-5000 – USA; 4 Charles M. Bowden Research Center, RDECOM, Redstone Arsenal, Alabama 35898-5000 – USA

## Abstract

In this paper, we report on the engineering and the realization of optically transparent graphene-based microwave devices using Chemical Vapour Deposition (CVD) graphene whose sheet resistance may be tailored down to values below 30 Ω/sq. In particular, we show that the process was successfully used to realize and characterize a simple, optically transparent graphene-based wire-grid polarizer at microwave frequencies (X band). The availability of graphene operating in a *quasi-metallic* region may allow the integration of graphene layers in several microwave components, thus leading to the realization of fully transparent (and flexible) microwave devices.

Chemical Vapour Deposition (CVD) graphene has been indicated as the ideal conducting material for future 2D technologies. However, the most current and lowest reported experimental values of its sheet resistance (around 1000 Ω/sq) literally invalidate any such prospects^1^. Microwave applications like for example optically transparent microwave antennas require graphene with sheet resistance in the *quasi-metallic* region^2^,^3^,^4^,^5^. For suspended monolayer graphene this means a sheet resistance lower than a critical value (η_0_/2, with η_0_ corresponding to the impedance of free space) where microwave absorption is expected to be maximum. Up to now, graphene properties in the microwave range have been studied by means of theoretical models^6^,^7^ in contexts including coplanar waveguides^8^,^9^, metallic rectangular waveguides^10^ and THz etalon measurements^11^. In most cases the sheet resistance falls in the *lossy-dielectric* region^3^ (values higher than η_0_/2), which strongly affects the performance of any microwave device that requires metallic components.

In this regard, the transport properties of CVD graphene are determined by both intrinsic and extrinsic factors such as (i) its polycrystalline nature and (ii) the lack of reproducible methods that would allow its transfer to substrates of technological interest without generating holes, cracks and ripples, especially on large areas. These defects, as well as grain boundaries, act as barriers to charge transport and result in sheet resistance higher than that of mechanically exfoliated micrometer graphene^12^. Indeed, the carrier mobility of CVD graphene (1000–3000 cm^2^ V^−1^ s^−1^) is approximately one order of magnitude lower than the carrier mobility of the mechanically exfoliated graphene, if measured inside a large single CVD graphene grain^13^. This suggests that the poor transport properties of CVD graphene are strongly correlated to the presence of structural defects in the carbon lattice (vacancies, substitutional impurities, and topological defects).

In theory, the use of multiple layers of CVD graphene acting as parallel resistances should amount to a simple method to lower the sheet resistance. However, experiments suggest that adding parallel CVD graphene layers does not necessarily decrease sheet resistance, since the partial contact or the presence of additional impurities (such as residual etchant and water molecules) contribute contact/series resistances between graphene layers^1^,^3^. Thus, the reduction of the sheet resistance of the individual graphene layers remains an important issue even when multilayer graphene is used.

Chemical doping is an effective way to enhance graphene sheet conductivity via the attachment of electron donors or acceptors. For example, the treatments with thionyl chloride (SOCl_2_) has been successfully used for hole-doping of graphene^14^, as well as other graphitic materials like reduced graphene oxides^15^ and carbon nanotubes^16^. The peculiarity of chemical doping by SOCl_2_ consists of the exploitation of the intrinsic defects in CVD graphene as enhanced reactive centers for the attachment of dopant functionalities. This provides the stability of covalent chemical doping without detrimental effects on material mobility due to the creation of new C-sp^3^ acting as charge scattering centers in the C-sp^2^ lattice.

We presently demonstrate that the combined use of SOCl_2_ chemical doping and multilayer graphene can reduce material sheet resistance down to 30 Ω/sq while optical transparency remains above 85%. Moreover, we show how this chemically optimized graphene may be used to realize and characterize a simple optically transparent graphene-based wire-grid polarizer at microwave frequencies by comparing its performance with its copper-based counterpart.

## Results

**Figure 1:**
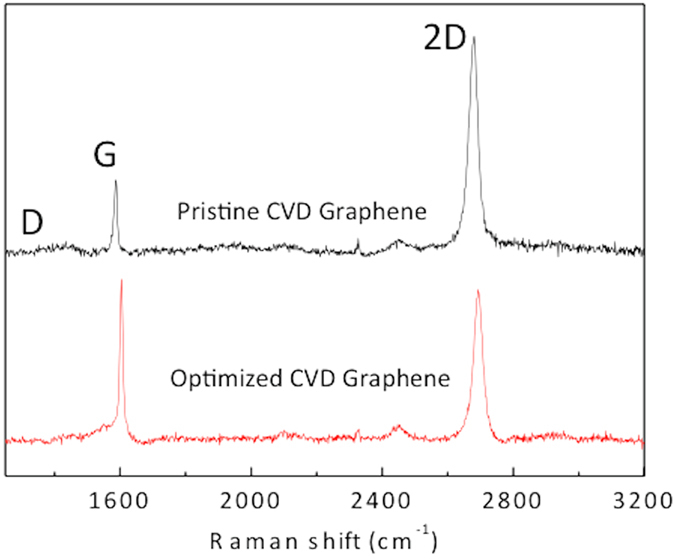
Comparison of the Raman spectra of monolayer CVD graphene before (black) and after (red) SOCl_2_ doping.

In Fig. 1 we report the Raman spectra of CVD graphene before and after SOCl_2_ treatment. The hole-doping effect is clearly demonstrated by the red-shift of both the G and 2D peaks and by the increase of the ratio between their intensities^17^. We note the absence of the D peak in the spectrum of the chemical doped graphene, which attests to the absence of any breaking in the C-sp^2^ lattice conjugation induced by the SOCl_2_ treatment. In general, the interaction between SOCl_2_ and graphitic materials is expected to occur by removing hydroxyl and carboxyl functionalities, and by introducing both covalently and ionically bonded chlorine atoms^14^,^16^. Indeed, since the SOCl_2_/graphene interaction as well as the resulting dopant functionalities on graphene is still the subject of discussion in the literature, the interaction with other SOCl_2_/graphene reaction products, including SO_2_, cannot be excluded^14^.

**Figure 2:**
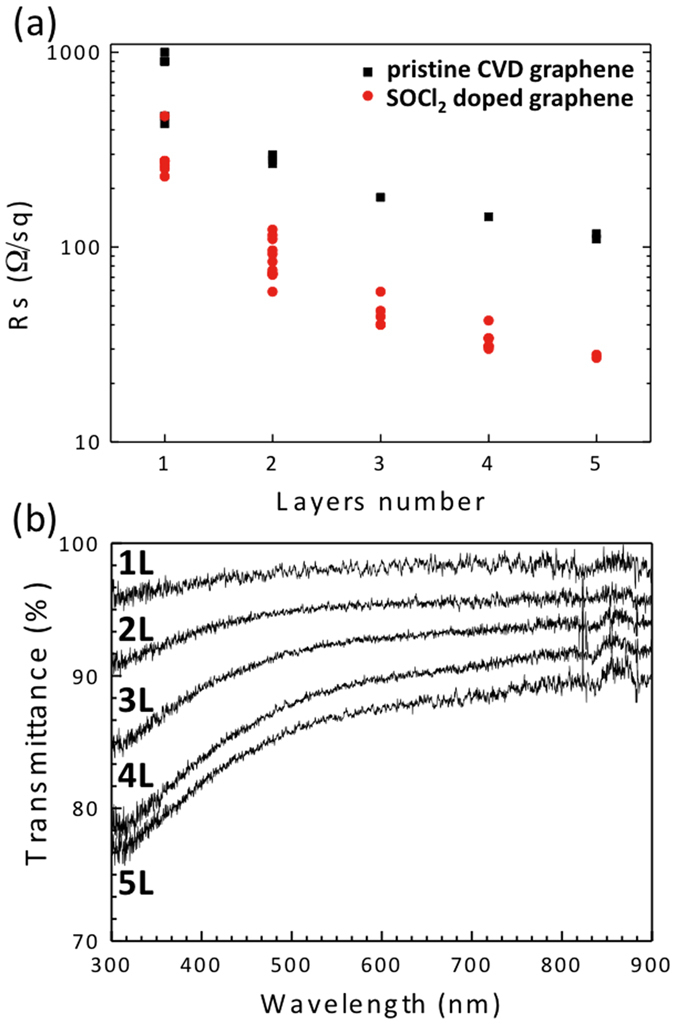
(**a**) Comparison of sheet resistance of 1–5 layers CVD graphene samples on glass substrate with (red circles) and without (black squares) SOCl_2_ doping. (**b**) Optical transmittance for 1–5 layer CVD graphene doped by SOCl_2_ treatment.

The effect of the combined use of SOCl_2_ chemical doping and multilayer graphene in terms of sheet resistance is shown in Fig. 2a. In going from the monolayer to five layers the sheet resistance R_s_ of pristine CVD graphene monotonically decreases and plateaus to values larger than 100 Ω/sq (black solid squares). In contrast, chemically doped graphene can overcome the low threshold limit with only two layers. The minimum sheet resistance value measured for chemically doped graphene is 27 Ω/sq, and was achieved with five-layer graphene samples. If additional graphene layers are added, the effect of series resistances between adjacent graphene layers becomes predominant, resulting in higher R_s_.

The optical transmittance of chemically doped graphene from one to five layers is reported in Fig. 2b. No differences were found between the transmittance values of pristine and chemically doped multilayer graphene. The higher absorbance found for three, four and five graphene layers are related mainly to residual impurities due to the transferal processes, and not to impurities introduced by SOCl_2_ treatment.

The possibility to operate with very-low sheet resistance samples (*quasi-metallic* graphene) allowed us to test the electromagnetic response of hole-doped graphene at microwave frequencies. In particular, we measured reflectance, transmittance, and absorbance of samples with different sheet-resistances. The measurements were carried out by means of a microwave setup consisting of a klystron connected to a WR90 rectangular waveguide (the waveguide supports only the TE_10_ mode). A slotted-line acquired the electromagnetic power (square law) with a spatial resolution of 1 mm in the rectangular waveguide at 9 GHz (the guide wavelength is λ_g_ = 48 mm, while the free-space wavelength is λ_0_ = 33 mm). Further details on the measurement setup are reported in the Supplementary Information (Paragraph SI1).

The analysis is based on the measurement of the standing waves that originate in the rectangular waveguide from the discontinuity at one end of the waveguide. In order to validate the experimental protocol we measured the reflection coefficient Γ when the waveguide is either shorted with a metallic plate or open-ended.

We began our analysis by measuring the response of glass samples covered by graphene sheets with different sheet resistances. The area covered by the doped graphene was about 3 cm × 2 cm, allowing total coverage of the WR90 waveguide cross-section.

We note that the thickness of the hole-doped graphene is of the order of 1 nm, corresponding to ~λ/10^6^, thus confirming the two-dimensional nature of graphene at microwave frequencies.

**Figure 3:**
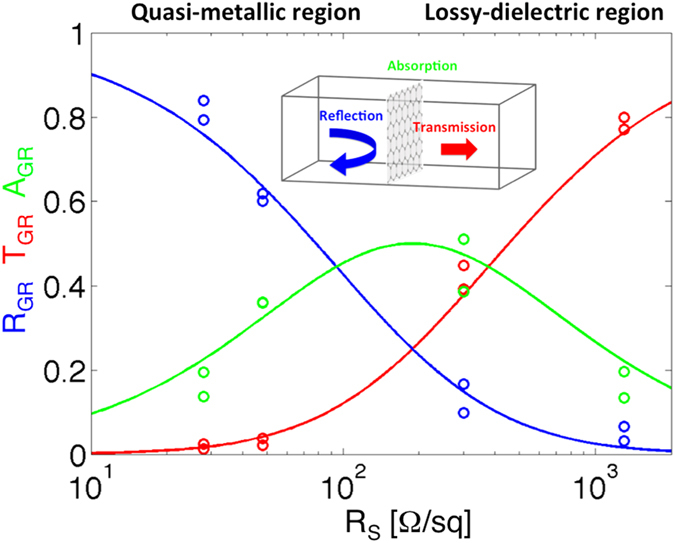
Analytical model (solid line) and experimental findings (circles) for the reflectance R_GR_ (blue), transmittance T_GR_ (red) and absorbance A_GR_ (green) when R_s_ is varied in the range 10 Ω/sq – 2 kΩ/sq. Please note that the x-axis is in logarithm scale. The maximum absorbance (obtained considering R_s_ = *η*_0_/2 where *η*_0_ is the vacuum impedance) separates the *quasi-metallic* region (R_s_ < *η*_0_/2) from the *lossy-dielectric* region (R_s_ > *η*_0_/2). The reflectance and transmittance were measured by means of a microwave setup operating at 9 GHz.

Figure 3 shows experimental reflectance, transmittance and absorbance of the hole-doped graphene when the sheet resistance varies from the *lossy-dielectric* region to the *quasi-metallic* range. The solid line refers to the analytical model described and implemented in the Methods (Analytical Model). The comparison between the analytical model and the experimental results shows very good agreement.

We emphasize that the maximum achievable absorption with the doped graphene sheet is equal to 50%, while the graphene in the *quasi-metallic* region acts as a metal by efficiently reflecting the impinging electromagnetic field. For example, when R_s_ = 27 Ω/sq the reflectance is larger than 80%. At the same time, the doped graphene sheet conductivity is independent over a wide frequency range (reported theoretically and experimentally in references [6, 7, 8, 9, 10, 11]), which makes the results reported in Fig. 3 also relatively frequency-independent, and may consequently be applied to other regions of the spectrum.

**Figure 4:**
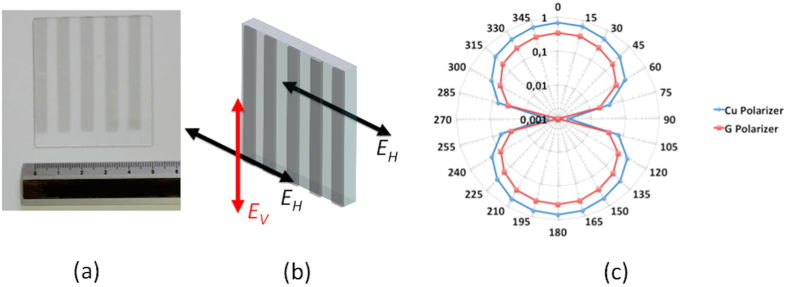
(**a**) Picture of the fabricated 4-layer-graphene-based wire-grid polarizer; (**b**) sketch of the graphene-based polarizer and its working principle. (**c**) Normalized transmittance (T_90_ – T_pol_)/T_90_ for the graphene-based polarizer (red curve) and the copper-based polarizer (blue curve) where T_90_ and T_pol_ are the transmittance at 90° and the polarizer transmittance, respectively.

The chemically optimized graphene was exploited to realize a graphene-based wire-grid polarizer, by superimposing four graphene stripes. Using this procedure we achieved a minimum sheet resistance of 50 Ω/sq (*quasi-metallic* region) in the stripes over an area of about 50 × 55 mm^2^ (Fig. 4a). The presence of *quasi-metallic* stripes allows the transmission of the electric field that is polarized along the horizontal direction (E_H_), while the electric field with vertical polarization (E_V_) is reflected (Fig. 4b).

In order to verify its behavior in terms of polarization, the graphene-based wire-grid polarizer was placed in between two horn pyramidal antennas with a mutual distance larger than that the Fraunhofer distance, thus allowing operation in the far-field (further details are reported in the Supplementary Information). The device was placed on a plastic goniometer (Δ⊖ step was set at 15°) and the transmitted signal was normalized with respect to a reference structure (glass substrate without graphene).

Figure 4 shows the normalized transmittance (T_90_ – T_pol_)/T_90_ of the polarizer T_pol_ with respect to transmission at 90° (T_90_). We adopted this normalization to emphasize the difference at 0° (reflected signal). In fact, the graphene-based wire-grid polarizer (red curve) shows a near-unity transmission at 90°.

We compared the graphene-based wire-grid polarizer with a wire-grid polarizer made with copper stripes of identical dimensions (5 mm width) and identical foot-print with respect to the graphene-based polarizer. The comparison reveals that the two polarizers only show a constant 3 dB difference over the entire angular range while their angular behavior is identical. This result is quite remarkable, considering that the copper layer is 35 μm thick, while graphene thickness is about 1 nm.

## Conclusion

In conclusion, we have detailed a chemical protocol for the optimization of graphene in terms of (i) doping stability, (ii) scalability to large areas, and (iii) reduction of the series resistances between graphene layers in multilayer graphene, thus achieving multilayer graphene with sheet resistance values below 30 Ω/sq.

This protocol allowed us to experimentally verify the optimized graphene behavior in terms of reflectance, transmittance, and absorbance of hole-doped graphene when the sheet resistance ranges from that of a *lossy-dielectric* to that of a *quasi-metal*.

Finally, the optimized graphene was exploited for the realization of an optically transparent graphene-based polarizer. The comparison between the graphene-based polarizer and its copper-based counterpart revealed that the two polarizers show only a constant 3 dB difference over the entire angular range while their angular behavior remains identical.

The possibility to operate in the *quasi-metallic* region with very-low sheet resistance graphene may allow the realization of microwave devices (e.g. microstrip patch antennas) and the dynamical tuning of their S-parameters over a wide-range. In this regard, graphene-based antennas with low-sheet resistance graphene may help overcome the limitation of low gain^2^,^3^,^4^.

## Methods

### CVD graphene growth and transferal

Graphene was grown by CVD on a 25 μm thick copper foils in a typical quartz tube CVD reactor at 1000 °C using CH_4_/H_2_ as precursors. The graphene was transferred onto corning glass substrate (treated by O_2_ plasma to improve graphene adhesion) by the thermal tape method. The copper was etched in a solution of ammonium persulfate. Multilayer graphene samples were fabricated by transferring of additional graphene layers onto graphene/glass substrates.

### Chemical doping

SOCl_2_ treatments were performed in a dry chamber by placing graphene/glass substrate and 1 mL of liquid SOCl_2_ (avoiding direct contact) at 105 °C for 60 min. Doping of multilayer samples was performed by repeating SOCl_2_ treatment after transferring and stacking each graphene layer.

### Electrical measurements

Sheet resistance measurements were carried out using four-point contacts geometry in the Van der Pauw configuration on a sampled area of 4 × 4 mm^2^ in air and at room temperature (further details on the measurement setup are reported in the Supplementary Information).

### Analytical model

Considering the boundary conditions at the interface z = 0, between two (semi-infinite) media (impinging from medium 1 to medium 2), that introduces a finite sheet conductivity σ_2D_, we can write:


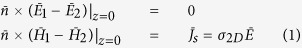


where *η*_1_ and *η*_2_ are the wave impedances in the two media, respectively, and 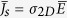 corresponds to Ohm’s law. When graphene is considered in the microwave regime, the graphene sheet conductivity can be approximated by the DC sheet conductivity, i.e. *σ*_2*D*_ ≈ *σ*_*DC*_ = 1/*R*_*s*_. Moreover, the sheet conductivity is independent over a wide frequency range, as found in references [6, 7, 8, 9, 10, 11]. At the same time, the graphene sheet may be considered as an interface (current sheet) between two media, since its thickness is ~λ/10^6^ at microwave frequencies (X-band). If we apply the boundary conditions for the electric and magnetic fields at the interface z = 0 we obtain:


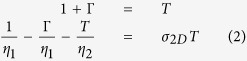


where Γ and T are the reflection and transmission coefficients, respectively.

Solving the Equation 2 yields to:


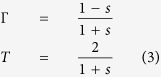


where we set 
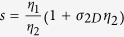
. Reflection and transmission coefficients allow calculation of reflectance R_GR_ and transmittance T_GR_, 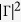 and 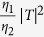, respectively, while absorbance A_GR_ may be evaluated as A_GR_ = 1 – R_GR_ – T_GR_. If we assume *η*_1_ = *η*_2_ = *η*_0_ (where η_0_ corresponds to the impedance of free space) it is possible to define two different regions for a single graphene sheet: the *lossy-dielectric* region 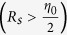
and the *quasi-metallic* region 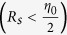, where 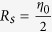 defines the sheet resistance that corresponds to the absorbance maximum.

## Additional Information

**How to cite this article**: Grande, M. *et al.* Optically Transparent Microwave Polarizer Based On Quasi-Metallic Graphene. *Sci. Rep.* **5**, 17083; doi: 10.1038/srep17083 (2015).

## Supplementary Material

Supplementary Information
