# Supplementary material for: Optically Transparent Microwave Polarizer Based On Quasi-Metallic Graphene
Source: Sci Rep. 2015 Nov 25;5:17083. doi: 10.1038/srep17083 (PMC4658520; doi:10.1038/srep17083)
Supplement: Supplementary Information [file srep17083-s1.pdf]

SUPPLEMENTARY INFORMATION

Optically Transparent Microwave Polarizer Based On Quasi-Metallic Graphene

Marco Grande[1,*,+], Giuseppe Valerio Bianco[2,*,+], Maria Antonietta Vincenti[3], Domenico de Ceglia[3], Pio Capezzuto[2], Michael Scalora[4], Antonella D'Orazio[1], and Giovanni Bruno[2]

[1] Dipartimento di Ingegneria Elettrica e dell'Informazione, Politecnico di Bari, Bari, 70125, Italy
[2] Istituto di Nanotecnologia – CNR-NANOTEC, Bari, 70126, Italy
[3] National Research Council, Charles M. Bowden Research Center, RDECOM, Redstone Arsenal, Alabama 35898-5000 – USA
[4] Charles M. Bowden Research Center, RDECOM, Redstone Arsenal, Alabama 35898-5000 – USA
* marco.grande@poliba.it; giuseppevalerio.bianco@cnr.it
[+] these authors contributed equally to this work

SI1. Microwave measurements. The analysis is based on the measurement of standing waves that originate in the rectangular waveguide WR90 due to the discontinuity at its end.

The standing waves allow measurement of the guide wavelength λ_g = 48 mm and determine the free-space wavelength λ_0 =33 mm. This corresponds to a working frequency f = 9 GHz (this frequency corresponds to the centre of the klystron operating frequency range).

We began our analysis by considering several glass slides covered with doped graphene with different sheet resistance R_s. The area covered by the doped graphene was about 3 cm x 2 cm, thus allowing total coverage of the waveguide cross-section.

The experimental reflection and transmission coefficients were retrieved by measuring the standing waves in the rectangular waveguide by means of a slotted-line (the measurements were carried out with a resolution of 1 mm, corresponding to about $\lambda_g / 50$) adopting the configurations sketched in Figure S1. The set-up in Figure 1(a) was adopted for the measurement of the reflection coefficients by varying the sheet resistance of the graphene sheets transferred onto the glass substrate. In contrast, the transmission coefficient was calculated by normalizing the transmission of the graphene sheets (T_G) with respect to the transmission of the glass reference sample (T_{ref}), as sketched in Figure 1(b). The transmission coefficient T is defined as the ratio between T_G and T_{ref}.

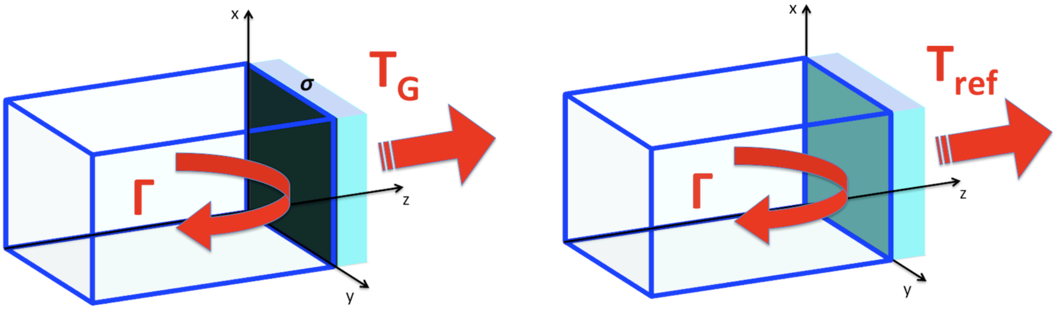

(a) (b)

Figure S1: Sketch of the measurement configurations (a) with and (b) without (reference sample) doped graphene sheet transferred on the glass substrate. The blue box represents the rectangular waveguide whilst the black thin layer indicates the graphene sheet with sheet conductivity σ.

Preliminary tests were performed on the open-ended waveguide and the waveguide capped with a shorting plate, resulting in excellent agreement with both theoretical and experimental results. The reflection coefficient of the open-ended waveguide is fully consistent with the experimental results reported in.[S1]

Finally, the transmittance for both polarizers (Cu-based and G-based ones) was measured by means of two pyramidal horn antennas placed about 35 cm apart (Figure S2). This distance satisfies the Fraunhofer distance equal to $d = \frac{2D^2}{\lambda}$ where D is the maximum dimension (in our case D = 7 cm).

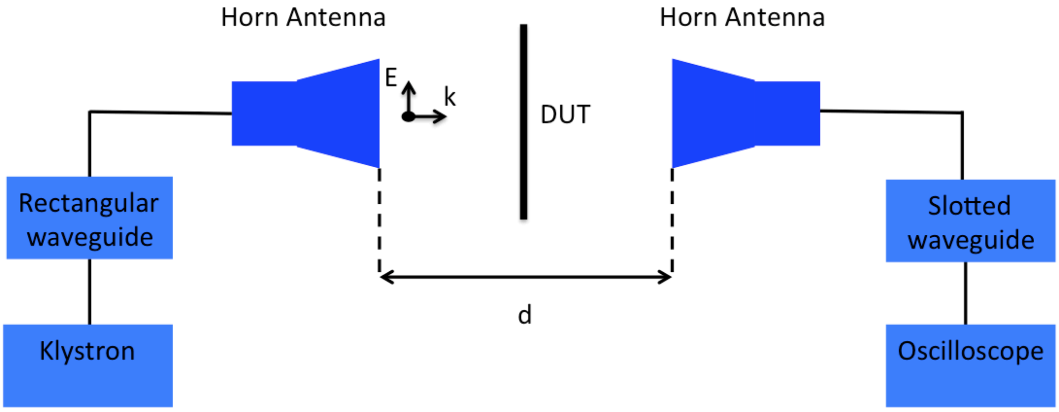

Figure S2: Illustration of the microwave setup for the transmittance measurements.

SI2. Analytical model. The boundary conditions at the interface z=0 (xy-plane in Figure S3) between two (semi-infinite) media (impinging from medium 1 to medium 2), that introduces a finite sheet conductivity σ_{2D}, may be written as:

$$\bar{n} \times (\bar{E}_1 - \bar{E}_2)\big|_{z=0} = 0$$
$$\bar{n} \times (\bar{H}_1 - \bar{H}_2)\big|_{z=0} = \bar{J}_s = \sigma_{2D}\bar{E} \qquad \text{(Eq. S1)}$$

where $\bar{J}_s = \sigma_{2D}\bar{E}$ is Ohm's law.

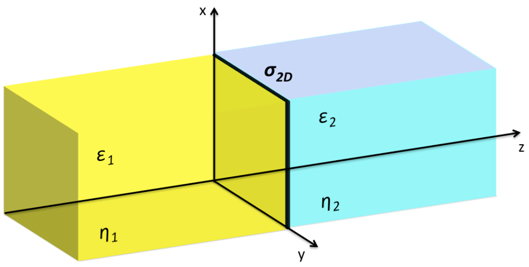

Figure S3: Finite conductive interface between two dielectric media.

At microwave frequencies the graphene sheet conductivity can be approximated by the DC sheet conductivity, *i.e.* $\sigma_{2D} \approx \sigma_{DC} = 1/R_s$. Moreover, the sheet conductivity is frequency-independent over a wide frequency range.[6,7-11] At the same time, the graphene sheet may be considered as an interface (current sheet) between two media, since its thickness is ~$\lambda/10^6$ at microwave frequencies (X-band).

If we apply the boundary conditions for the electric and magnetic fields at the interface z=0 we obtain:[S2]

$$1 + \Gamma = T$$
$$\frac{1}{\eta_1} - \frac{\Gamma}{\eta_1} - \frac{T}{\eta_2} = \sigma_{2D}T \qquad \text{(Eq. S2)}$$

where Γ and T are the reflection and transmission coefficients, respectively, and η_1 and η_2 are the wave impedances in the two media.

Solving the Equation (S2) yields to:

$$\Gamma = \frac{1-s}{1+s}$$
$$T = \frac{2}{1+s} \qquad \text{(Eq. S3)}$$

where we set $s = \frac{\eta_1}{\eta_2}\left(1 + \sigma_{2D}\eta_2\right)$. Reflection and transmission coefficients are used to derive reflectance R_{GR} and transmittance T_{GR}, equal to $|\Gamma|^2$ and $\frac{\eta_1}{\eta_2}|T|^2$, respectively, while absorbance A_{GR} can be evaluated as A_{GR}=1-R_{GR}-T_{GR}.

If we assume $\eta_1 = \eta_2 = \eta_0$ it is then possible to identify two different regimes for the single graphene sheet: the lossy-dielectric regime $\left(R_s > \frac{\eta_0}{2} \right)$ and the quasi-metallic regime $\left(R_s < \frac{\eta_0}{2} \right)$, where $R_s = \frac{\eta_0}{2}$ defines the sheet resistance corresponding to the absorbance maximum, as shown in Figure S4.

The system sketched in Figure S3 was also simulated by means of a 3D-FEM-based code (COMSOL) as shown in Figure S4 (markers). The comparison reveals a near-perfect match between the two approaches.

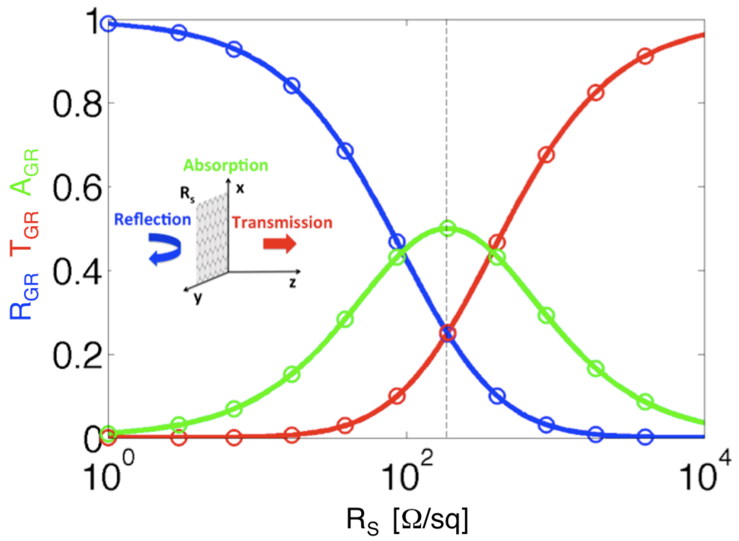

Figure S4: Reflectance (blue curve), transmittance (red curve), and absorbance (green curve) when R_s is varied from 1 Ω to 10 kΩ/sq. The dashed line separates the quasi-metallic region from the lossy-dielectric region. The *x*-axis is in logarithmic scale. (inset) Sketch of the doped graphene interface.

We verified that the same analytical and numerical procedures could be efficiently extended to the rectangular waveguide, where the waveguide impedances (Z_{TE}) replace the wave impedances for the two media. Figure S5 shows the reflectance of the doped graphene layer on a 0.7 mm-thick substrate placed at the output of the WR90 metallic waveguide. In particular, the plot shows that the reflectance of the doped graphene is almost constant over the range 0-150 Ω/sq when the permittivity of the substrate is varied from 2 to 6: the reflectance variation is only to 1% when R_s = 100 Ω/sq regardless of substrate permittivity. This result confirms the quasi-metallic behaviour of the doped graphene.

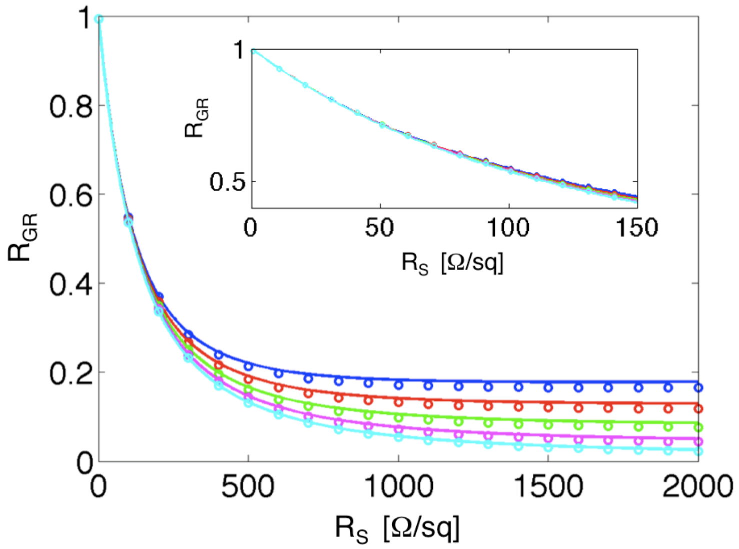

Figure S5: Reflectance of the doped graphene when the permittivity of the substrate (0.7 mm) is varied from 2 (cyan) to 6 (blue). The solid lines and the markers refer to the analytical model and 3D-numerical simulations (COMSOL). (inset) Zoom of the plot when R_s is varied from 1 to 150 Ω/sq.

SI3. Sheet resistance setup. Sheet resistance measurements were carried out using four-point contacts geometry in the Van der Pauw configuration on a sampled area of 4 x 4 mm^2 in air and at room temperature. The reported values of graphene sheet resistance are the average values deriving from several measurements performed by directly probing the graphene at different locations on each sample.

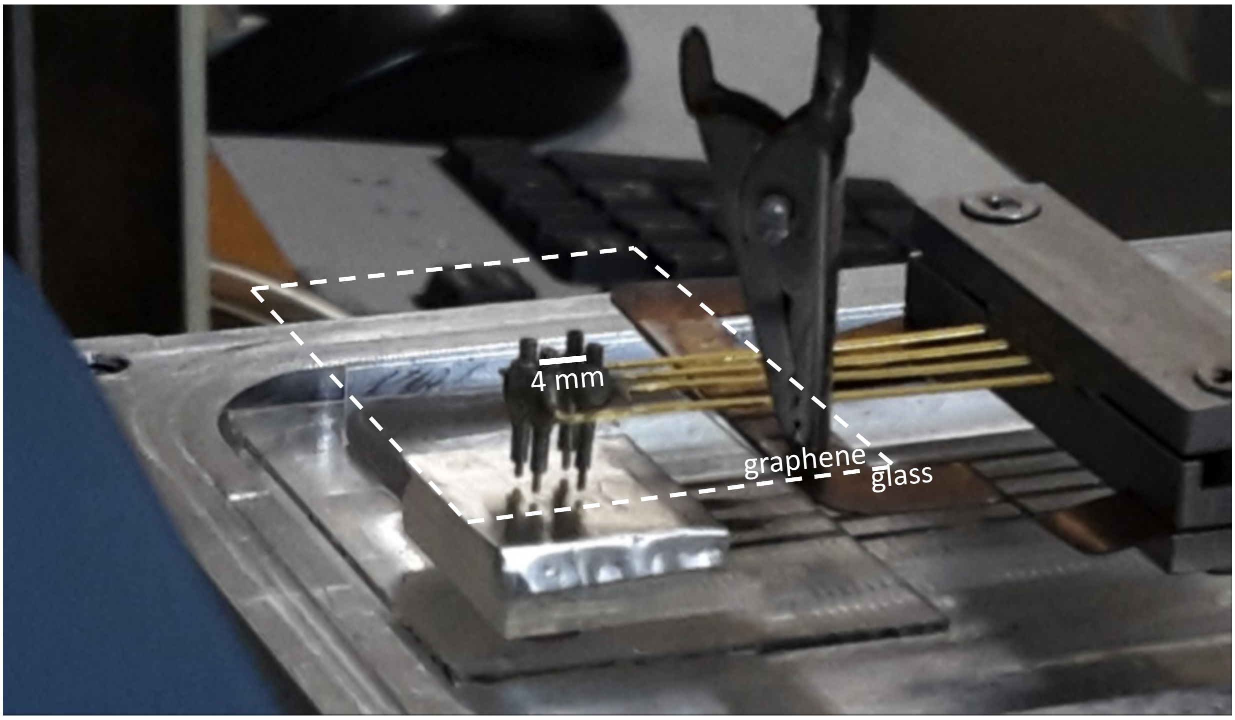

Figure S6: Picture of the sheet resistance measurement setup (MMR Technologies, Inc).

References

S1. Yaghjian, A. D. Approximate Formulas for the Far Field and Gain of Open-Ended Rectangular Waveguide. *IEEE Transactions on antennas and propagation* **32**, 378-384 (1984).

S2. Pozar, D. M. Microwave Engineering, Wiley, 1997.
